# Fantastic Voyage: Influenza

**DOI:** 10.3201/eid1503.080669

**Published:** 2009-03

**Authors:** Julian W. Tang

**Affiliations:** National University Hospital, Singapore

**Keywords:** influenza, nosocomial, airborne, aerosol, infection, cancer, infection control, negative pressure, another dimension

Time to go! Time to go!
An influenza virus,
Hiding in saliva, buried in a cell,
Antibodies and T-cells are coming.
Get out! But how?
Induce a sneeze? Kick-start a cough?
Ah yes, here we go …
Aahhh-choo! Freedom at last!
Where are we?
A quick look around
A hospital? I see children,
Very thin, sick children,
Must be the cancer ward.
No B cells or T cells—a virus paradise!
Let’s travel, find a breeze and float.
Where’s my next victim?
My previous young doctor host
Walking quickly away
Head down, embarrassed
Scolded by a nurse—where was his mask?
Too busy and careless, poor fool
He still serves me well, dragging me into his wake.
Here I go, following and floating
A door nearby opens—negative pressure!
In I go, but on a cancer ward,
This should be positive pressure!
To keep bugs out, not draw them in.
I cannot complain
All good for me, for now I can see
A young girl with leukemia
Sitting in bed, watching TV
Laughing, inhaling, bringing me close.
But wait, what’s this? Her mother!
Opening the toilet door,
Even greater negative pressure in there.
And worse, wet surfaces glistening inside,
Cleaned by her mother with chlorhexidine.
No! Not yet! Not now! I’m so close!
Being pulled in. No escape. Falling, falling …
The young girl is laughing. Now her mother is too.

